# Decreased Glycogenolysis by *miR-338-3p* Promotes Regional Glycogen Accumulation Within the Spinal Cord of Amyotrophic Lateral Sclerosis Mice

**DOI:** 10.3389/fnmol.2019.00114

**Published:** 2019-05-07

**Authors:** Chunyu Li, Qianqian Wei, Xiaojing Gu, Yongping Chen, Xueping Chen, Bei Cao, Ruwei Ou, Huifang Shang

**Affiliations:** Department of Neurology, National Clinical Research Center for Geriatrics, West China Hospital, Sichuan University, Chengdu, China

**Keywords:** ALS, glycogen, glycogenolysis, PYGB, *miR-338-3p*

## Abstract

Metabolic dysfunction is a hallmark of age-related neurodegenerative diseases, including amyotrophic lateral sclerosis (ALS). But the crosstalk between metabolic alteration and disease progression in ALS is still largely unknown. Glycogen, a branched polymer of glucose residues, is universally recognized as the energy reserve of the central nervous system (CNS), where its aberrant accumulation instigates neurodegeneration. Glycogen was reported to be accumulated in both CNS and visceral organs of SOD1^G93A^ mice, a well-known ALS model, and contributes to the pathological process of ALS. However, the accumulative patterns and mechanisms are not well elucidated. Here, we provide extensive evidence to demonstrate that glycogen accumulated in the lumbar spinal cord of ALS mice along with the disease progression, but not in the motor cortex. This regional accumulation of glycogen was caused by deteriorated glycogenolysis, which was triggered by decreased glycogen phosphorylase, brain form (PYGB). Moreover, *miR-338-3p*, an elevated miRNA in the spinal cord of SOD1^G93A^ mice, directly targeted PYGB and was responsible for the decreased glycogenolysis and subsequent glycogen accumulation. Our work is helpful for better understanding of of of metabolic dysfunctions in ALS and provides novel targets for the therapeutic intervention in the future.

## Introduction

In the central nervous system (CNS), metabolic homeostasis is required to integrate external and internal stimuli for information processing (Bélanger et al., 2011; Camandola and Mattson, 2017). Impairment of central metabolism occurs in age-related neurodegenerative diseases, hallmarked by mitochondrial dysfunction, oxidative stress, and protein aggregation (Lin and Beal, 2006; Liu et al., 2017). All these impairments bring about damage to neurons and glia, and contribute to the demise of neurons (Lin and Beal, 2006). In amyotrophic lateral sclerosis (ALS), an age-dependent neurodegenerative disease (Kwiatkowski et al., 2009), a growing body of evidence suggests that metabolic imbalance within the brain and spinal cord is responsible for the initiation and progression of motor neuron degeneration (Ioannides et al., 2016; Veyrat-Durebex et al., 2016; Abdel-Khalik et al., 2017). However, exact mechanisms underlying the metabolic discrepancy in pathological processes of ALS remain poorly understood.

Glucose is the obligatory energy substrate for CNS, where it is fully oxidized to produce ATP (Steinbusch et al., 2015). After entering into cells, glucose is phosphorylated and utilized through different metabolic pathways, including glycolysis/tricarboxylic acid (TCA) cycle for energy production (Goyal et al., 2017), the pentose phosphate pathway (PPP) for antioxidative capacity (Nóbrega-Pereira et al., 2016), and glycogenesis for energy storage (Duran and Guinovart, 2015). Glycogen, a branched polymer of glucose residues, is an important storage form of energy mainly in liver and muscle (Adeva-Andany et al., 2016). Glycogen metabolism is regulated by two enzymes, glycogen synthase (GYS) and glycogen phosphorylase (PYG). GYS is responsible for the glycogen synthesis (glycogenesis), including two isoforms, namely GYS1 expressed in most tissues (Browner et al., 1989) and GYS2 in the liver (Nuttall et al., 1994). PYG is the rate-limiting enzyme for glycogen degradation (glycogenolysis), with three tissue-specific isoforms in mammalians, namely PYGM in the muscle, PYGL in the liver, and PYGB in the brain (Duran and Guinovart, 2015; Mathieu et al., 2016). Glycogenesis and glycogenolysis are precisely regulated processes that contribute to glycogen homeostasis.

Although glycogen in CNS is much lower than that in the muscle or liver (Nelson et al., 1968), it is exclusively localized in astrocytes for inductive releasing by neurotransmitters (Bak et al., 2018). As for neurons, glycogen level is very low even compared with astrocytes, because over-accumulation of glycogen induces neuronal death (Vilchez et al., 2007). Neurons have the enzymatic machinery for synthesizing glycogen (Saez et al., 2014). However, its activity is kept restrained by a series of well-coordinated mechanisms (Vilchez et al., 2007). In neurodegenerative diseases, such as Alzheimer’s disease (Inoue et al., 1996), Parkinson’s disease (Trivedi et al., 2003), and Lafora disease (Wang et al., 2013; Duran et al., 2014), glycogen is known to accumulate in neurons. This aberrant accumulation of glycogen causes glucotoxicity and impairs neuronal functions, leading to neurodegeneration.

As a fatal neurodegenerative disease, ALS is marked by prominent metabolic dysfunction, however, how glycogen metabolism is altered and contributes to disease progression of ALS is not clear. A previous report described significant accumulation of glycogen in both CNS and visceral organs of SOD1^G93A^ mice, a well-known ALS animal model (Dodge et al., 2013). But the evidence of glycogen accumulation in ALS still needs to be confirmed, and its mechanisms need to be explored as well. In this study, we demonstrate that glycogen is extraordinarily accumulated in the spinal cord of ALS mice, but not in the motor cortex. This regional accumulation of glycogen is caused by deteriorated glycogenolysis, which is controlled by the *miR-338-3p*/PYGB axis. Our work provides deep insights into the metabolic dysfunctions in the progression of ALS and affords a novel target for the therapeutic interventions of ALS in the future.

## Materials and Methods

### Reagents and Chemicals

The following reagents were acquired from commercial sources and used following the manufacturer instructions: miRNeasy Mini Kit (Qiagen), miScript II reverse transcription kit (Qiagen), miScript miRNA polymerase chain reaction (PCR) Arrays (Qiagen), Dulbecco’s modified Eagle’s medium (DMEM, GIBCO), trypsin (GIBCO), Fetal Bovine Serum (FBS, GIBCO), anti-GYS1 antibody (3886, Cell Signaling Technology; Palamiuc et al., 2015), anti-PYGB antibody (12075-1-AP, Proteintech; Takezawa et al., 2014), and RNAiMAX (Thermo Fisher Scientific).

### SOD1^G93A^ Transgenic Mice

B6SJL-Tg (SOD1^G93A^) mice were purchased from the Jackson Lab (Bar Harbor, ME, USA). Animal care and all experimental procedures were compliant with the Laboratory Animal Care Guidelines approved by the Animal Care and Use Committee of Sichuan University West-China Hospital. We used SOD1^G93A^ mice at the following stages, namely pre-symptomatic stage (8–10 weeks, Pre), symptomatic stage (14–15 weeks, hereafter Onset) and end stage (19–20 weeks, hereafter End) (Weydt et al., 2003).

### Glycogen Measurement

Glycogen content of motor cortex and spinal cord tissues was measured by the glycogen assay kit (BioVision) as described (Oe et al., 2016). Briefly, different stages of SOD1^G93A^ mice and wild type littermates (*n* = 5 at least per point) were transcardially perfused with phosphate-buffered saline (PBS). Cerebral motor cortex and lumbar spinal cord were dissected immediately after perfusion and weighed afterward. Tissues were then rapidly homogenized with 200 μl ddH_2_O for 1 min on ice. Homogenates were boiled for 10 min to inactivate enzymes and were then centrifuged at 18,000 rpm for 10 min before the supernatant was collected. The supernatant of boiled sample was used for glycogen quantification based on protocols provided by the Biovision Assay Kit.

To measure glycogen content of Neuro2a cells, glycogen was firstly digested by amyloglucosidase, and released glucose was assessed as described (Singh et al., 2012). Briefly, cells were lysed in 30% potassium hydroxide (KOH) and boiled at 100°C for 20 min. A small aliquot of the sample was saved for protein estimation using the BCA method for quantification, and the rest was spotted onto a filter paper. The paper was washed in ice-cold 70% ethanol three times and each time for 10 min, dried at 37°C, and then incubated in amyloglucosidase for 2 h. The released glucose was measured using a glucose assay kit (Sigma), and the content of glycogen is presented as the amount of released glucose per milligram of total protein.

### Periodic Acid-Schiff (PAS) Staining

The lumbar spinal cord at different stages of SOD1^G93A^ mice and wild type littermates (*n* = 5 per point) were extracted following PBS and paraformaldehyde (PFA, 4% in standard PBS) perfusion, and stored in 4% PFA at 4°C for 24 h. The tissue fixation, embedding and sectioning were followed as standard protocol (Zeller, 2001). For PAS staining, the lumbar spinal cord sections were deparaffinized and hydrated in decreasing concentrations of ethanol. The sections were then oxidized in 0.5% periodic acid solution for 5 min and rinsed in distilled water, placed in Schiff reagent for 15 min, and then washed in lukewarm tap water for 5 min. The sections were then counterstained with Mayer’s hematoxylin for 1 min, washed in tap water for 5 min, dehydrated, and mounted in synthetic resin (Acrytol; Leica Microsystems). After being dried for 24 h, the tissue section was visualized using an Olympus IX 81 (Olympus, Tokyo, Japan) microscope.

### RNA Extraction and Real-Time Quantitative PCR Assay

The total RNA extraction and real-time quantitative PCR (RT-qPCR) for mRNA and miRNA were performed following standard procedures as previously described (Li et al., 2017). Briefly, total RNA, including miRNA, was extracted and collected using miRNeasy Mini Kit (Qiagen) according to the manufacturer’s protocol. Then 1 μg of total RNA was reverse-transcribed into cDNA (Thermo Fisher Scientific) and RT-qPCR was performed with SYBR Green PCR Master Mix using the Bio-Rad iQ5 system. The relative gene expression was normalized to internal control as ACTB (β-actin). Primer sequences for the target genes were as follows, and data analysis was performed using the 2^−ΔΔCt^ method.

**Table:** 

	Forward (5′–3′)	Reverse (5′–3′)
ACTB	GAGACCTTCAACACCCCAGC	ATGTCACGCACGATTTCCC
Gys1	GAACGCAGTGCTTTTCGAGG	CCAGATAGTAGTTGTCACCCCAT
Gys2	ACCAAGGCCAAAACGACAG	GGGCTCACATTGTTCTACTTGA
Pygb	CCGCGACTACTTCTTCGCTC	CAACCCCAACTGATAAGTGGC

For RT-qPCR of miRNA, both inverse transcription and qPCR were carried out according to the manufacturer’s protocol (Qiagen). RNU6 was used as an internal control and all the primers for miRNAs were purchased from Ribobio (China). All results were calculated and expressed as 2^−ΔΔCt^.

### Cell Culture and Treatment

For *in vitro* experiments, we chose the neuroblastoma cell line Neuro2a obtained from American Type Culture Collection (ATCC). Neuro2a cells were grown at 37°C, 5% CO_2_ with a constant humidity environment. Dulbecco’s Modified Eagle’s Medium (DMEM, Gibco) containing 10% of FBS (Gibco), 100 U/mL penicillin, 100 mg/mL streptomycin (Invitrogen) was used to maintain the cells. Cells around 80%–90% confluency were passaged to maintain the running culture. Cells were transfected *miR-338-3p* mimics, inhibitors or scramble sequences (Ribobio) using RNAiMAX (Invitrogen) according to the manufacturer’s instructions.

### Luciferase Reporter Assay

For the luciferase reporter assay, wildtype and mutant 3′UTR of *Pygb* were cloned into the pMIR-REPORT luciferase vector (Ambion, USA) by *Xba*I and *EcoR*I sites, and the reconstituted plasmids were named as pWT and pMUT, respectively. All inserted or mutated sequences were confirmed by sequencing. Neuro2a cells were plated in 96-well plates and co-transfected with mimics or negative control (miR-NC) with pWT or pMUT by RNAiMAX according to the manufacturer’s instructions (Thermo Fisher Scientific). Luciferase activity was measured in cell lysates at 48 h after transfection and normalized to the Renilla luciferase activity.

### Protein Extraction and Western Blots

Western blot analyses were performed according to our previous protocols (Li et al., 2017). Total proteins were extracted with lysis buffer (2% SDS with protease and phosphatase inhibitors) and protein concentration of each extract was measured by BCA Protein Assay kit (Thermo Scientific Pierce) according to the instructions. An equal amount of denatured proteins (~20 μg) were loaded into and separated by SDS-PAGE and then transferred onto PVDF membranes following standard procedures. The membranes were blocked with 5% skimmed milk in TBST (TBS with 0.1% Tween 20, pH 7.4) for 1 h at room temperature on a rocker and then incubated with proper antibodies diluted in TBST (1:1,000) at 4°C, overnight. The membranes were washed three times with TBST for 10 min each time, and the membranes were incubated with proper secondary antibodies diluted in TBST (1:10,000 for both the goat anti-rabbit and goat anti-mouse IgG antibodies) for 2 h at 37°C. The membranes were washed three times with TBST at room temperature and each wash lasted for 10 min. Proteins were then detected with ECL reagent (Thermo Scientific/Pierce, Rockford, IL, USA) and the membranes were exposed to film (Kodak). Resulting films were scanned, and optical densities were quantified using ImageJ software.

### Statistical Analysis

All quantitative results were presented as mean ± SEM unless otherwise stated. Statistical tests were performed by GraphPad Prism 6 software. *P* values were calculated using the two-tailed unpaired *t*-test, one-way ANOVA test, followed by a Turkey *post hoc* test to find mean differences between groups. The *P* value 0.05 (*), 0.01 (**) and 0.001 (***) was assumed as levels of significance for the statistic tests carried out.

## Results

### Progressive Accumulation of Glycogen in the Spinal Cord of ALS Mice

**Figure 1:**
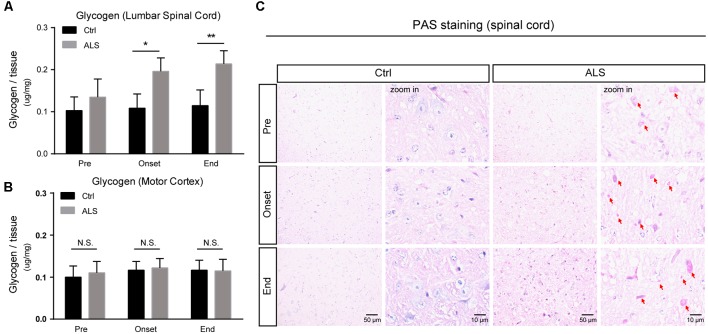
Progressive accumulation of glycogen in the lumbar spinal cord of amyotrophic lateral sclerosis (ALS) mice. **(A,B)** Bar diagram showing glycogen levels in the lumbar spinal cord and motor cortex of SOD1^G93A^ transgenic mouse (ALS mice) during disease progression, compared with wildtype littermates. Data are shown as the mean ± SEM values and are the result of six independent experiments. Statistical analysis was performed by using two-tailed unpaired *t*-test. *P < 0.05, **P < 0.01 and N.S., no statistical significance. **(C)** Periodic acid-schiff (PAS) staining images showing PAS-positive glycogen granules (pointed by red arrows) in the anterior horn of lumbar spinal cord of ALS mice (scale bar, 50 μm or 10 μm). Pre, presymptomatic stage; Onset, symptomatic stage; End, end stage.

Although glycogen level is extremely low in CNS, many studies have established that glycogen acts to provide supplemental substrates for energy consumption and supports learning and memory. In CNS, glycogen is mainly restricted to astrocytes, and its ectopic accumulation in neurons leads to neuronal death. However, how improper glycogen metabolism is associated with ALS remains largely unknown. Here, to investigate the role of glycogen in ALS progression, we firstly examined glycogen contents in SOD1^G93A^ mice (hereafter ALS mice), an extensively used animal model for ALS studies (Gurney et al., 1994). Biochemical results showed that glycogen content was obviously increased in the lumbar spinal cord of ALS mice (Figure 1A). Interestingly, it’s noticed that the glycogen level was not increased in the motor cortex of ALS mice (Figure 1B), implying a local glycogen accumulation in the spinal cord.

To confirm this regional glycogen accumulation, we performed periodic acid-schiff (PAS) staining to visualize glycogen distribution and accumulation. Histochemical images showed remarkable glycogen particles in the spinal cord of ALS mice, from symptom onset to end stages (Figure 1C). Taken together, our data support that the accumulation of glycogen emerges in ALS mice, indicating that glycogen aberrancy may be highly correlated with ALS onset and progression.

### Decreased Glycogenolysis, but Not Glycogenesis, Is Responsible for Glycogen Accumulation in the Spinal Cord of ALS Mice

**Figure 2:**
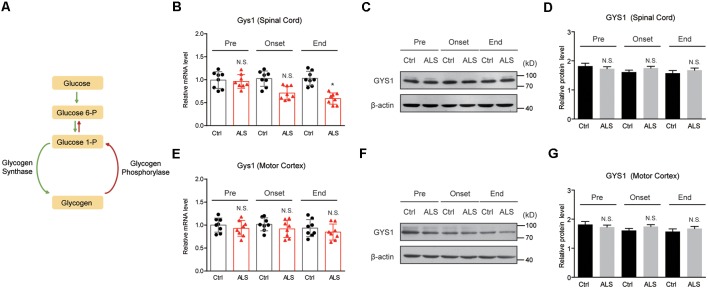
GYS1 expression is not dramatically changed in ALS mice.** (A)** Schematic graph of glycogen metabolism. **(B–D)** Real-time quantitative polymerase chain reaction (RT-qPCR) and western blot results showing the mRNA **(B)** and protein levels **(C,D)** of GYS1 in the spinal cord of ALS mice, compared with controls. Data are shown as the mean ± SEM values and are the result of eight independent experiments. Statistical analysis was performed by using two-tailed unpaired *t*-test. N.S., no statistical significance. *P < 0.05. **(E–G)** RT-qPCR and western blot results showing no significant difference in both mRNA **(E)** and protein levels **(F,G)** of GYS1 in the motor cortex of ALS mice, compared with controls. Data are shown as the mean ± SEM values and are the result of eight independent experiments. Statistical analysis was performed by using two-tailed unpaired *t*-test. N.S., no statistical significance.

Glycogen metabolism is tightly monitored by glycogen synthesis (glycogenesis) and glycogen degradation (glycogenolysis), which are controlled by the activities of GYS and PYG (Figure 2A). To explore the mechanisms of glycogen accumulation, we firstly examined GYS expressions in ALS mice. Quantitative PCR results showed that the mRNA level of *Gys1*, the major form of GYS in the CNS, was not altered in the spinal cord of ALS mice at the pre-symptomatic and symptomatic stage, but decreased at end stage (Figure 2B). Western blot results further confirmed that the protein level of GYS1 was not dramatically changed during disease progression in ALS mice (Figures 2C,D). Moreover, we found that neither mRNA nor protein level of GYS1 was altered in the motor cortex of ALS mice, consistently with that in the spinal cord (Figures 2E–G). All these data indicate that glycogen accumulation in ALS mice is not due to increased glycogenesis.

**Figure 3:**
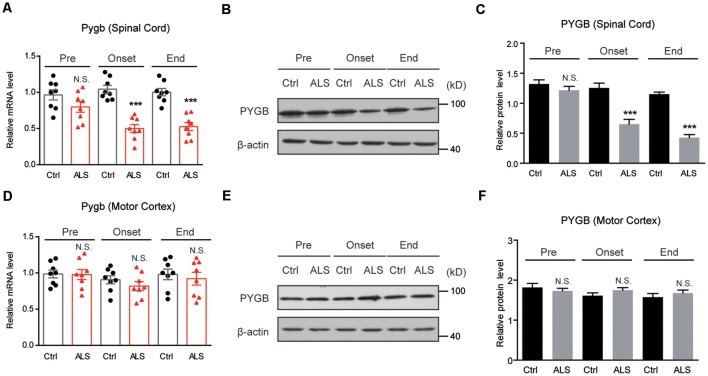
PYGB expression is decreased regionally in the lumbar spinal cord of ALS mice. **(A–C)** RT-qPCR and western blot results showing that the mRNA **(A)** and protein levels **(B,C)** of PYGB are decreased in the spinal cord of ALS mice, compared with controls. Data are shown as the mean ± SEM values and are the result of eight independent experiments. Statistical analysis was performed by using two-tailed unpaired *t*-test. N.S., no statistical significance, and ***P < 0.001. **(D–F)** RT-qPCR and western blot results showing no significant difference in both mRNA **(D)** and protein levels **(E,F)** of PYGB in the motor cortex of ALS mice, compared with controls. Data are shown as the mean ± SEM values and are the result of eight independent experiments. Statistical analysis was performed by using two-tailed unpaired *t*-test. N.S., no statistical significance.

Next, we examined PYGB (glycogen phosphorylase in the brain) expression in ALS mice. By RT-qPCR, we found that the mRNA level of *Pygb* was decreased in the spinal cord of ALS mice along with disease onset (Figure 3A). Moreover, we found that the protein level of PYGB was decreased, consistent with mRNA changes in ALS mice (Figures 3B,C). Interestingly, we noticed that neither mRNA nor protein levels of PYGB were altered in the motor cortex of ALS mice (Figures 3D–F), suggesting that PYGB was regionally decreased in the spinal cord. Taken together, our data reveal that decreased glycogenolysis is responsible for glycogen accumulation in the spinal cord of ALS mice.

### *miR-338-3p* Targets PYGB to Inhibit Glycogenolysis in the Spinal Cord of ALS Mice

**Figure 4:**
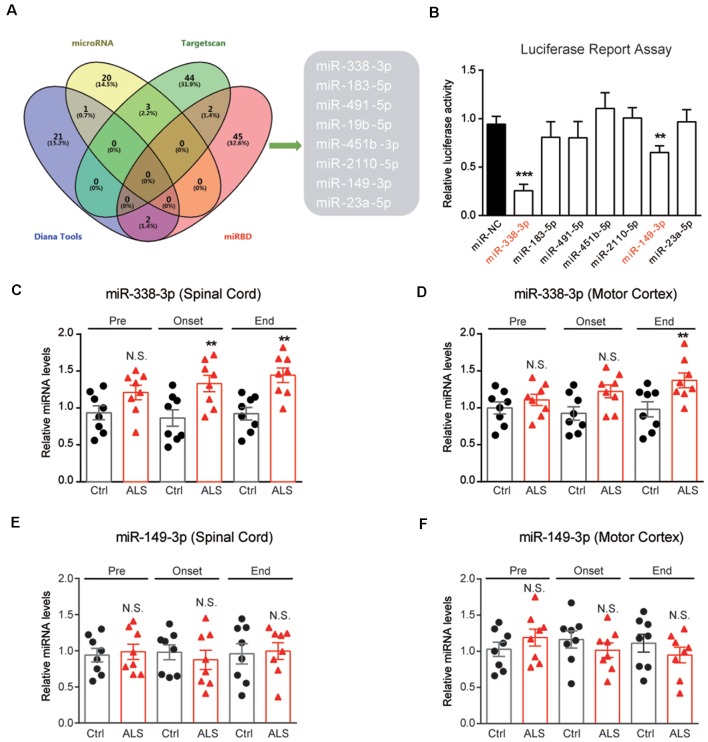
Screening of miRNAs targeting PYGB. **(A)** Overlap examinations performed on four different miRNA prediction databases by VENNY. Eight miRNAs were found, namely *miR-338-3p*, *miR-183-5p*, *miR-491-5p*, *miR-19b-5p*, *miR-451b-5p*, *miR-2110-5p *, *miR-149-3p*, and *miR-23a-5p*. **(B)** Luciferase reporter assay showing *miR-338-3p and miR-149-3p* may directly bind to the 3′UTR of PYGB. Data are shown as the mean ± SEM values and are the result of three independent experiments. Statistical analysis was performed by using one-way ANOVA test followed by a Turkey *post hoc* test. **P < 0.01 and ***P < 0.001. **(C–F)** RT-qPCR results showing the changes of *miR-338-3p and miR-149–3p* in the lumbar spinal cord and motor cortex. Data are shown as the mean ± SEM values and are the result of six independent experiments. Statistical analysis was performed by using two-tailed unpaired *t*-test. N.S., no statistical significance, and **P < 0.01.

To investigate the mechanisms of PYGB downregulation in ALS mice, we performed bioinformatic predictions to screen miRNAs targeting PYGB. By screening databases of MicroRNA^1^, Targetscan^2^, Diana Tools^3^ and miRDB^4^, we found that *miR-338-3p*, *miR-183-5p*, *miR-491-5p*, *miR-19b-5p*, *miR-451b-5p*, *miR-2110-5p*, *miR-149-3p*, and *miR-23a-5p* may target PYGB, and be accountable for its post-transcriptional modifications (Figure 4A). Furthermore, the luciferase reporter assay confirmed that *miR-338-3p* and *miR-149-3p* are plausible to target PYGB (Figure 4B).

Because PYGB is regionally decreased in the spinal cord of ALS mice, it is very likely that miRNAs targeting PYGB should also alter regionally. Therefore, we inspect the expressions of *miR-338-3p* and *miR-149-3p* in ALS mice. Results showed that *miR-338-3p* was increased in the spinal cord of ALS mice along with disease onset (Figure 4C). Moreover, *miR-338-3p* was not increased in the motor cortex of ALS mice at early and middle stages of the disease but increased at the end stage (Figure 4D). As for *miR-149-3p*, we found that it’s not significantly altered in both the spinal cord and the motor cortex of ALS mice (Figures 4E,F). Taken together, the regional increase of *miR-338-3p* was compatible with PYGB decrease, suggesting that *miR-338-3p* may be responsible to regulate PYGB in ALS mice.

**Figure 5:**
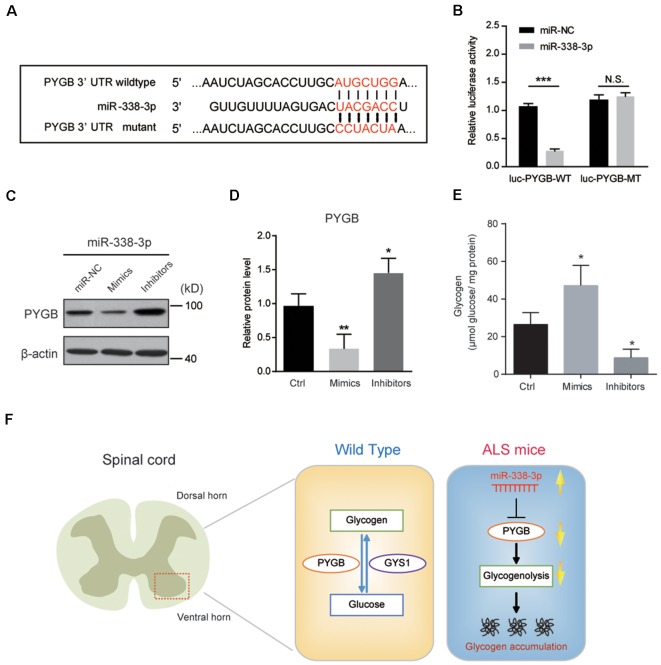
*miR-338-3p* targets PYGB to inhibit glycogenolysis in Neuro2a cells. **(A)** Sequence analysis of *miR-338-3p* mature miRNA binding with the 3′-UTR of PYGB. **(B)** Luciferase reporter assay showing reduction of luciferase activity in N2a cells compared with wild-type PYGB 3′UTR (luc-PYGB-WT) plasmids, but not in the seed region mutant PYGB 3′UTR (luc-PYGB-MT) plasmids. Data are shown as the mean ± SEM values and are the result of three independent experiments. Statistical analysis was performed by using two-tailed unpaired *t*-test. N.S., no statistical significance, and ***P < 0.001. **(C,D)** Western blots and quantifications showing the expression of PYGB in Neuro2a cells transfected with *miR-338-3p* mimics, inhibitors or scramble sequence (100 nM, respectively). Data are shown as the mean ± SEM values and are the result of four independent experiments. Statistical analysis was performed by using one-way ANOVA test followed by a Turkey *post hoc* test. *P < 0.5 and **P < 0.01. miR-NC: miRNA negative control (scramble sequence). **(E)** Relative glycogen levels in Neuro2a cells transfected with *miR-338-3p* mimics, inhibitors or scramble sequence (100 nM, respectively). Data are shown as the mean ± SEM values and are the result of four independent experiments. Statistical analysis was performed by using one-way ANOVA test followed by a Turkey *post hoc* test. *P < 0.5. **(F)** A schematic model to show that regional glycogen accumulation in the lumbar spinal cord of ALS mice, which is caused by increased *miR-338-3p* and decreased PYGB.

To confirm whether *miR-338-3p* directly targets PYGB expression, we carried out a luciferase reporter assay in Neuro2a cells co-transfected with wild type or mutant 3′UTR of PYGB and *miR-338-3p* as the sequence analysis indicated (Figure 5A). We found that *miR-338-3p* could bind to the wild type 3′-UTR of PYGB, but not the mutant (Figure 5B), indicating that *miR-338-3p* could directly bind to PYGB. Besides, biochemical results showed that the protein level of PYGB was obviously decreased by *miR-338-3p* overexpression (mimics) and increased by its inhibition (inhibitors; Figures 5C,D). Finally, we found that *miR-338-3p* mimics could increase glycogen contents in Neuro2a cells, and vice versa (Figure 5E). Thus, our results suggest that *miR-338-3p* directly targets PYGB to regulate glycogenolysis and glycogen contents in the spinal cord of ALS mice.

## Discussion

Many studies established that metabolic deviation is a pivotal cause of the onset and progression of aging-related neurodegenerative diseases, such as ALS (Ioannides et al., 2016; Marini et al., 2016; Szelechowski et al., 2018). However, the crosstalk between altered metabolism and disease progression in ALS remains largely unexplored. It has been established that aberrant accumulation of glucose metabolites, especially glycogen, impairs neuronal functions and causes neurodegenerative diseases (Dodge et al., 2013; Duran et al., 2014; Duran and Guinovart, 2015). However, mechanisms underlying the glycogen accumulation is not clear. In this study, we reveal the direct link between glycogen accumulation and ALS progression. We show that glycogen, the major form of glucose storage, is aberrantly accumulated in the lumbar spinal cord of ALS mice, but not in the motor cortex. This regional accumulation of glycogen is caused by deteriorated glycogenolysis, which is triggered by decreased PYGB. Moreover, *miR-338-3p*, an elevated miRNA in the spinal cord of ALS mice, directly targets PYGB and is responsible for decreased glycogenolysis and subsequent glycogen accumulation (Figure 5F).

Glycogen is now accepted as an energy reserve of the brain, although it is at much lower concentrations than that in liver and muscle (Bélanger et al., 2011; Mergenthaler et al., 2013). Under physiological conditions, glycogen is exclusively localized in astrocytes, which could be mobilized by neuronal activity to provide an energy source for neurons (Bak et al., 2018). Neurons tightly keep glycogen synthesis inactive to maintain few glycogen contents (Vilchez et al., 2007). Pathological accumulation of glycogen would initiate neuronal death. The potential link between glycogen accumulation and neurodegenerative diseases was revealed by aggregation of glycogen particles in Huntington’s disease (Rai et al., 2018) and ALS (Dodge et al., 2013), but the accumulative patterns and mechanisms are not well elucidated. Here, we provide definitive evidence to show that glycogen accumulates in the lumbar spinal cord of ALS mice, not in the motor cortex. Furthermore, we demonstrate that it is decreased glycogenolysis, but not glycogenesis, which is responsible for this regional glycogen accumulation. For ethnic limitations, we could not examine the glycogen accumulation in the autopsy brain and spinal cord tissues of ALS patients. However, results from Dodge et al. (2013) reveal that obvious glycogen accumulation is incontrovertible in ALS patients (Dodge et al., 2013), which is consistent with our findings in ALS mice. Therefore, we conclude that glycogen accumulation could be appreciated as a novel metabolic feature of ALS.

Glycogen homeostasis is precisely regulated by glycogenesis and glycogenolysis. In glycogenesis, GYS is responsible for glycogen synthesis. In mammalians, GYS has two isoforms, namely GYS1 and GYS2. GYS2 is expressed only in the liver, whereas GYS1 is widely expressed in most tissues including CNS (Browner et al., 1989). Here, we show that neither the mRNA nor protein level of GYS1 is altered in the spinal cord of ALS mice, suggesting that glycogenesis may not increase. On the other hand, glycogenolysis is controlled by PYGB in CNS (Mathieu et al., 2016). We found that PYGB expression is obviously decreased in the spinal cord of ALS mice locally. Therefore, we propose that it is the decreased glycogenolysis that causes glycogen accumulation in ALS mice, whereas glycogenesis keeps intact. It is noted that there are three isoforms of PYG in mammalians, including PYGB, PYGL and PYGM. As in the CNS, the major functional isoform is PYGB (Mathieu et al., 2016), which is dynamically regulated by *miR-338-3p*. And we also found that neither PYGL nor PYGM was monitored by *miR-338-3p* (data not shown), suggesting that *miR-338-3p* is specifically targeted to PYGB to modulate glycogen metabolism in the spinal cord. Our findings uncover the critical role of glycogenolysis in the maintenance of glycogen homeostasis in CNS.

How is PYGB expression is regulated in ALS? By bioinformatic analysis, we found that miRNA-mediated post-transcriptional regulation controls PYGB expression. We showed that *miR-338-3p* binds to the 3′UTR of PYGB, and thus inhibits PYGB expression. Interestingly, we found that *miR-338-3p* is intensely increased in the spinal cord of ALS mice, which is consistent with previous studies (De Felice et al., 2014), indicating that *miR-338-3p* expression is highly associated with ALS progression. Moreover, we demonstrate that *miR-338-3p* inhibition by inhibitors could effectively increase PYGB expression in Neuro2a cells, indicating *miR-338-3p* as a potential target for intervention of glycogen accumulation in ALS mice. Although our mechanism findings of *miR-338-3p* on PYGB are generated from Neuro2a cells *in vitro*, these results provide novel clues for illuminating metabolic dysfunctions in neurodegenerative diseases, and afford opportunities for consequent studies *in vivo*.

In conclusion, our work identifies a regional accumulation of glycogen in the spinal cord of ALS mice, which is caused by decreased glycogenolysis *via* diminished PYGB expression. Our findings provide deep insights into the metabolic dysfunctions in the progression of ALS and afford novel targets for therapeutic interventions of ALS in the future.

## Ethics Statement

B6SJL-Tg (SOD1^G93A^) 1Gur/J mice were purchased from the Jackson Lab (Bar Harbor, ME, USA). Animal care and all experimental procedures were compliant with the Laboratory Animal Care Guidelines approved by the Animal Care and Use Committee of Sichuan University West-China Hospital. We used SOD1^G93A^ mice at following stages, namely presymptomatic stage (8–10 weeks, Pre), symptomatic stage (14–15 weeks, hereafter Onset) and end stage (19–20 weeks, hereafter End).

## Author Contributions

HS and CL conceived and designed the research. CL collected, analyzed and interpreted the data and drafted the manuscript. CL, QW and XG performed the experiments. HS, YC, XC, BC and RO revised the article critically. All authors approved the manuscript.

## Conflict of Interest Statement

The authors declare that the research was conducted in the absence of any commercial or financial relationships that could be construed as a potential conflict of interest.

## Footnotes

^1^http://www.microrna.org/microrna/home.do

^2^http://www.targetscan.org

^3^http://diana.imis.athena-innovation.gr/DianaTools/index.php

^4^http://mirdb.org/miRDB/

## Abbreviations

ALS: amyotrophic lateral sclerosis
CNS: central nervous system
GYS: glycogen synthase
PYG: glycogen phosphorylase
PYGB: glycogen phosphorylase brain form.
